# Quit attempts among current tobacco users attending the outpatient department of Dr Yusuf Dadoo district hospital, South Africa

**DOI:** 10.4102/safp.v62i1.5128

**Published:** 2020-08-17

**Authors:** Arlette I. Bokoro, Elizabeth Reji, Olufemi B. Omole

**Affiliations:** 1 Division of Family Medicine, University of the Witwatersrand, Johannesburg, South Africa

**Keywords:** tobacco users, outpatient, family medicine, tobacco quit attempts, motivations and barriers of cessation

## Abstract

**Background:**

Implementing effective tobacco cessation programmes requires an understanding of the factors that influence quit attempts in a given context. In this study, we explored these factors among current tobacco users attending the outpatient department (OPD) of Dr Yusuf Dadoo Hospital, South Africa.

**Methods:**

In a cross-sectional study involving 275 tobacco users, a researcher-administered questionnaire collected socio-demographic, clinical, tobacco use and quit attempt information. Outcomes of data analysis included the proportion of participants who made quit attempts, the motivations and barriers, and the factors significantly associated with quit attempts.

**Results:**

The mean age of the participants was 46.5 years. Most of them were black (61.8%), male (65.8%), and had at least one chronic disease (52.7%) – of which 55.2% had a cardiovascular disease. About 87% of participants smoked cigarettes while 10% used snuff. Most participants made a quit attempt in the past year (74%), perceived it important to quit (92.0%) and felt confident to do so (75.0%). Health concern was the most common motivation for making a quit attempt, while advice from a healthcare provider was the least. Stress and cravings were the top two barriers to make a quit attempt. In regression analysis, being married was the only factor independently associated with making a quit attempt (odds ratio [OR]: 2.13; confidence interval [CI]: 1.17–3.86, *p* = 0.01).

**Conclusion:**

Most participants showed readiness to quit. However, healthcare professionals failed to leverage on participants’ motivations about their health to scale up the provision of quit advice to promote smoking cessation.

## Introduction

Despite tobacco use being a major risk factor of premature morbidity and mortality^1^ and a progressive tobacco control programme in South Africa, an estimated 37% and 8% of men and women, respectively, still used one form of tobacco product or the other in 2016.^2^

The adverse health outcomes attributed to tobacco use are mostly because of cigarette smoking and its effects on increasing the risk of non-communicable diseases, such as chronic obstructive pulmonary disease, cancers, stroke, ischaemic heart and peripheral vascular diseases.^3^

Quitting tobacco use at any stage is beneficial and associated with a reversal or slowing down of the pathological process.^4^ However, tobacco use cessation is challenging – of about 72% of adult smokers who would like to quit, only 24% would try and less than 10% will succeed in each attempt, cumulatively resulting in a 50% lifetime chance of quitting.^5^ Similarly, of 56% of smokeless tobacco (SLT) users who desire to stop, up to 42% will be unable to do so.^6^ These low quit rates are attributed to the addictive nature of nicotine and the difficulties in coping with nicotine withdrawal symptoms.

The addictive nature of nicotine and the uphill task of quitting make it imperative that tobacco users be motivated to make quit attempts at every opportune contact because doing so cumulatively increases the odds of success over time.^7^ However, initiating and having a successful quit attempt not only depends on the degree of nicotine dependence and the number of past quit attempts but also on motivations and barriers to quit attempts and sociodemographic, environmental, behavioural and health-related factors.^8,9,10^ While these factors have been documented in studies from developed countries, literature is sparse on whether the same holds true for developing countries, including South Africa – especially amongst tobacco users who are already at significant risk of adverse health outcomes. Knowing this is important for developing tailor-made interventions that could improve the quit rates in South Africa.

The outpatient department (OPD) of a district hospital is critical to provide ambulatory care to patients, a significant proportion of whom attend for the management of chronic non-communicable diseases.^11^ As tobacco use is a major risk factor for adverse health outcomes, the promotion of tobacco use cessation, especially smoking cessation, is a clinical and policy imperative. To do so effectively, local data that provide an understanding of the cessation journey in this population are needed. The aim of this study was to describe the pattern of quit attempt and determine factors that are associated with making a quit attempt amongst current tobacco users in the OPD of Dr Yusuf Dadoo Hospital (DYDH), West of Johannesburg.

## Methods

### Design and research setting

This cross-sectional study was conducted amongst patients 18 years and older attending the DYDH, Krugersdorp, west of Johannesburg, South Africa between May and August 2016. At the time the study was conducted, the hospital has 245 beds and caters for patients referred from 44 fixed primary care clinics, eight mobile clinics, four satellite clinics and three maternity obstetric units. The OPD was run by four medical doctors, two clinical associates (physician assistants) and 10 nurses.

### Study population, recruitment and sampling

According to the district health information system, about 23 892 patient visits were recorded at the OPD in 2014, excluding emergency cases. Assuming each patient attended the OPD once a month, an estimated 1991 patients were seen in the OPD in 2015. Assuming a target population of 1991, a sampling error of 5%, a confidence interval (CI) of 95% and tobacco use prevalence of 26%,^12,13^ the required sample size was determined to be 258. This was adjusted upwards to 275 to compensate for potential incomplete or missing data.

A pilot study was conducted in April 2016 with 10 patients in the OPD of the DYDH after obtaining ethics clearance to test the study feasibility and identify logistical problems. The patients involved in the pilot study were excluded from the final research sample and analysis.

**FIGURE 1:**
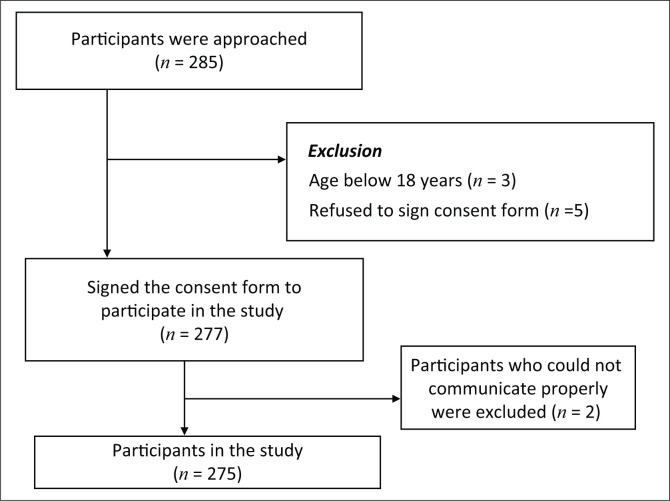
Flow chart showing the process of selecting the study participants.

All patients who were current tobacco users attending the OPD were eligible and were invited to participate in the research. Figure 1 presents the sampling flow chart.

Advertising posters inviting tobacco users to participate in the study were placed in the OPD and health promoters were requested to also inform patients about the study during the daily health talk in the reception. Patients who were willing to participate in the study were given information leaflets explaining the purpose of the study. The health promoters explained the study to those interested and who could not read.

Recruitment was done by staff nurses who enquired about all patients’ tobacco use status while measuring their vital signs. Those who reported using tobacco were directed to the first author’s consultation room, where they were briefed about the nature and scope of the study. Then, the researcher obtained informed written consent from patients who agreed to participate in the study. Patients who declined to participate were returned to their original position in the queue to get attended to by any of the other doctors in the OPD according to usual processes of care. Those who agreed to participate had their usual care followed by the questionnaire with the first author. Participants were recruited consecutively from Monday to Friday during work hours until the sample size was achieved.

### Data collection

A researcher-administered questionnaire written in English was used to collect data. This questionnaire was developed based on the findings of literature reviewed, especially the Global Adult Tobacco Survey.^14,15^ The first section of the questionnaire (Q1–Q8) collected information on participants’ socio-demographic characteristics and clinical problems. The second section (Q9–Q32) collected information on patterns of tobacco use and cessation attempts, motivations and barriers.

Patients who consented to participate in the study were first attended by the first author for their clinical needs and thereafter they were interviewed using the questionnaire. A trained research assistant proficient in the three prominent local languages (Sotho, Zulu and Afrikaans) translated to English for participants who did not speak or understand English. After completing the questionnaire, participants who required medication were directed to the OPD dispensary. A sticker was placed on the patient’s file to avoid resampling. The first author collected all the completed questionnaires daily and secured them. Data were later captured into a computer file with password protection.

### Data analysis

Data were captured onto an Microsoft Excel sheet and transferred to STATA release 14 for coding and analysis. Descriptive statistics were used to describe participants’ characteristics and their tobacco use patterns. The proportions of tobacco users who reported ever and quit attempt in the past one year were determined. Motivations and barriers to quit attempts reported by participants were also described. Group comparisons between those who had ever attempted to quit and those who had never tried to quit were performed regarding socio-demographic characteristics and tobacco use pattern using chi-square and *t*-tests, as applicable. Logistic regression analyses were used to determine factors significantly associated with quit attempts. Statistical significance was set at *p* < 0.05.

### Ethical considerations

Ethical approval to conduct the study was obtained from the Human Research Ethics Committee (HREC – Medical) of the University of the Witwatersrand (clearance number: M160208). Permission was obtained from the Chief Executive Officer of the DYDH before conducting the study. Informed consent was obtained from all the patients before their participation in the study.

## Results

A total of 275 patients participated in the study and their characteristics are shown in Table 1. The mean age of the participants was 46.5 years. Most participants were black (61.8%), male (65.8%), unemployed (53.83%), unmarried (61.09%), had completed Grade 12 education (48.73%) and had a chronic disease (52.7%). About 63% (172) attended the OPD for acute problems. Of those with chronic medical conditions, 55.17% (80) had cardiovascular diseases (CVD) as shown in Table 2.

**TABLE 1:** Participants’ socio-demographic characteristics.

Characteristics	Frequencies (*n* = 275)		Mean ± SD
	*n*	%	
**Age (years)**	-	-	46.5 ± 15.2
**Sex**			
Male	181	65.82	-
Female	94	34.18	-
**Population group**			
Black	170	61.82	-
White	98	35.64	-
Coloured	7	2.55	-
**Marital status**			
Unmarried (single, divorced and/or widowed)	168	61.09	-
Married (married and/or cohabiting)	107	38.91	-
**Level of education**			
No formal education	21	7.64	-
Primary	54	19.64	-
Secondary	134	48.73	-
Matric	48	17.45	-
Tertiary	18	6.55	-
**Employment status**			
Employed	127	46.18	-
Unemployed (includes students and pensioners)	148	53.82	-

SD, standard deviation.

**TABLE 2:** Participants’ clinical characteristics.

Variables	Frequencies	
	*n*	%
**Presence of chronic conditions (*n* = 275)**		
Participants with no chronic conditions	130	47.27
Participants with chronic conditions	145	52.73
**Types of chronic conditions that were present in tobacco users (*n* = 145)**		
Cardiovascular diseases	80	55.17
Infectious diseases	24	16.55
Mental health disorders	19	13.10
Respiratory diseases	14	9.66
Endocrine diseases	8	5.52
**Reason for the clinical encounter (*n* = 275)**		
Acute health problems	172	62.55
Follow-up visit	99	36.00
Administrative needs	4	1.45

Eighty-seven percent (238) of participants were cigarette smokers, while 10.2% (28) used snuff. Of those who smoked cigarette, the mean cigarettes per day (CPD) was 10 while the mean duration of smoking was 23 years. Black participants, those employed and without chronic conditions were more likely to report CPD of < 20, while white people, pensioners and those with chronic conditions were more likely to report a CPD of > 20. Alcohol was the most used substance with tobacco (87.17%) as shown in Table 3.

**TABLE 3:** Tobacco use patterns.

Variables	*n*	%	Mean	SD
**Types of tobacco products used (*n* = 275)**				
Cigarettes	238	86.55	-	-
Snuff	28	10.18	-	-
Hookah (water-pipe)	1	0.36	-	-
Other (‘roll your own’/bidi)	8	2.91	-	-
**CPD (*n* = 246)**				
< 10	150	61.00	-	-
11–19	76	31.00	-	-
20–29	9	4.00	-	-
≥ 30	11	4.00	-	-
**Duration of smoking (years)**			23	14.65
< 10	52	21.00	-	-
11–20	71	29.00	-	-
> 20	123	50.00	-	-
**No. of snuff dips per day (*n* = 28)**				
< 3 times	10	35.71	-	-
> 3 times	18	64.29	-	-
**Other substances used with tobacco (alcohol, dagga, nyaope, tik, and heroin) (*n* = 109)**				
Alcohol only	95	87.17	-	-
Dagga only	10	9.17	-	-
Nyaope only	2	1.83	-	-
More than one of the above	2	1.83	-	-
**Quit attempts (*N*= 275)**				
Ever made a quit attempt	203	73.82	-	-
Never made a quit attempt	72	26.18	-	-
Made a quit attempt in the last 1 year	112	55.17	-	-

SD, standard deviation; CPD, cigarettes per day.

Cigarette smokers were more likely to attend the OPD for chronic diseases compared to snuff users (odds ratio [OR] = 3.90, CI: 1.53 – 9.90, *p* = 0.004).

Seventy-four per cent of participants (203) reported ever making a quit attempt. Of these, 55% (112) did so in the last one year. The mean number of quit attempts in the last one year was 2.3.

Ninety-two percent (253) of participants reported that it was important to quit tobacco use, while 75% (207) felt confident in quitting.

Motivations and barriers to quit attempts are shown in Table 4. Health concerns were the most common motivation for making a quit attempt (64%), while the least common motivation was receiving quit advice from a healthcare professional (1.97%). On the other hand, stress (37.43%) and cravings (33.49%) were the most common barriers to making a quit attempt.

**TABLE 4:** Motivations and barriers to making a quit attempt.

Variables	Frequency (*n* = 203)	
	*n*	%
**Motivations**		
Health concerns	130	64
Desire to quit	55	27
Financial constraints	34	17
Advice from friend or relative	17	8
Religious reasons	7	3
Starting a family	5	2
Work recommendations	4	2
Advice from healthcare worker	4	2
**Barriers**		
Stress	76	37
Cravings	68	33
Feeling of addiction	55	27
Lack of willpower	24	12
Alcohol	22	11
Enjoyment/pleasure	21	10
Friend or relatives who smoke	21	10
Emotional traits, such as anger and aggression	5	2
Traditional reasons	3	1
Headache	3	1

Note: The total percentage of these factors was not equal to 100% as participants could select more than one factor.

In logistic regression analysis, being married was the only variable associated with quit attempt (OR: 2.13; CI: 1.17 – 3.86, *p* = 0.01) as shown in Table 5.

**TABLE 5:** Relationship between sociodemographic factors and quit attempt using logistic regression.

Variables	Odds ratio	Confidence interval	*p*
White population group	1.66	1.00–2.75	0.05
Marital status	2.13	1.17–3.86	0.01
Employment status	1.06	0.87–1.30	0.50

## Discussion

The findings of this study indicate that most tobacco users were light cigarette smokers and showed readiness to quit tobacco use. Although most participants were mostly motivated by health concerns (and least by advice from a healthcare professional) to make a quit attempt, stress and cravings were the top two reported barriers. These findings reiterate those of previous studies^9,16^ and in addition highlight poor healthcare professionals’ response to patients’ health concerns regarding their tobacco use behaviour. Hence, there is a need for health system-wide changes that will equip and prompt healthcare professionals to explore patient’s tobacco use status, their readiness to quit, their motivations and barriers, and their past experiences of quit attempts.

The finding that advice from healthcare providers was the least common motivation to make a quit attempt is worrisome given that tobacco use is an established risk for non-communicable diseases,^17,18,19^ and half of the participants with a chronic disease in this study had a CVD. Promoting quit attempts should have therefore been a priority task during these clinical encounters. This is more so that most participants were light smokers and quit advice from a healthcare professional would have increased the odds of making a quit attempt, which in turn increases the odds of successful cessation.^20^ Considering that many healthcare professionals in South African primary care are not confident in providing evidence-based tobacco cessation treatments,^16^ training on tobacco and health is a health sciences education and professional development imperative.

Without being screened first as a tobacco user, a patient cannot be offered quit advice indicating that the scaling up of offer of quit advice would have public health impact only if a functional screening system is in place. However, literature suggests that as few as 12.9% of patients are being screened in South African primary care.^21^ To address this gap, tobacco use status needs to be assimilated as part of the clinical vital signs such that it prompts healthcare providers to provide quit advice as part of the tasks of the clinical encounter. On another note, offering quit advice should have been viewed by healthcare professionals as a clinical imperative in the study setting because most participants were motivated by concerns for their health and this should have been explored by a patient-centred clinician. Such an exploration offers ‘teachable moments’ during which tobacco cessation advice and counselling may be offered, especially that ‘teachable moments’ have been shown to increase patients’ acceptance of advice offered by the healthcare professional.^17^

Most of the participants in this study reported readiness to quit and made multiple quit attempts in the last 12 months, but failed. This finding is similar to the findings of a previous study in a health district adjacent to the current study setting^21^ and reflects the difficulties in overcoming nicotine addiction.^15^ Showing readiness to quit does not guarantee success and while counselling may be more effective when readiness is high, the level of nicotine dependence needs to be explored. For tobacco users who are dependent on nicotine, treatment must include a combination of non-pharmacological and pharmacological interventions as this combination increases the likelihood of successful quit attempt almost three times.^22,23^

The more the number of previous quit attempts, the more the likelihood of successful cessation.^18^ Therefore, healthcare professionals need to explore previous quit attempt experiences and leverage on these to provide support. Since the barriers reported in this study (Table 4) are consistent with the literature,^9^ it is important that withdrawal symptoms, psychosocial stressors and other roadblocks experienced during previous quit attempts are explored and used to map a more effective way forward for future quit attempts.

Most smokers in this study are light smokers (mean CPD of 10), which is in agreement with the findings of previous South African studies, indicating that most would not need pharmacotherapy to successfully quit.^24^ Non-pharmacological interventions such as brief advice, counselling and behavioural therapy are effective and particularly scalable in resource-constrained health systems such as in South Africa.^4,25^ However, a significant proportion of healthcare providers in South Africa do not feel confident in providing tobacco cessation treatments.^16,26^ This is a key gap in clinical practice and medical training curricula, and reiterates the need for inclusion of tobacco cessation treatments training in academic curricula and clinical training of health professions students. For scalability, ancillary categories of healthcare professionals (health promoters and community healthcare workers) should be targeted for training such that some of the non-pharmacological treatments may be task-shifted to them.

The dual use of alcohol and tobacco products negatively impacts quit attempts as alcohol consumption is one of the predictors of tobacco cessation failure, and smokers are likely to smoke more when drinking.^27,28,29,30,31^ Therefore, not only should the use of other substances be explored amongst tobacco users but also counselling offered to address alcohol misuse to increase the success rate of quit attempts.^22^

The correlates of quit attempts in this study align with the literature regarding the role of sociodemographic disparities in tobacco cesation.^8,23^ Being married or having a partner avails tobacco users social support and this may in turn improve the ability to cope with nicotine withdrawal symptoms, increasing the chances of successful cessation.^23^ Thus, efforts should be made by healthcare providers to recruit social support resources for tobacco users during cessation attempts, as this may assist in mitigating stressors and other barriers, and reduce the risk of relapse.

This is a cross-sectional study and the generalisation of findings may be limited by the temporal nature of this research design. The use of self-report could have introduced some elements of information and recall biases into the study, with potential for under- or over-reporting. The recruitment of consecutive patients could also have introduced some elements of sampling bias. Notwithstanding these potential limitations, this study provides an understanding of quit attempts amongst tobacco users who are already at health risk and deepens our understanding of the cessation journey of tobacco users in a typical South African district hospital. The findings have implications for clinical management, promotion of quit attempts and tobacco cessation treatment, both in this and similar settings.

## Conclusion

Tobacco users in this ambulatory hospital setting reported readiness for quitting tobacco use and made quit attempts, mostly motivated by health concerns. Considering the benefits of tobacco cessation and that most of the participants had a form of chronic disease, the finding that ‘advice from a healthcare professional’ was the least common motivation for making a quit attempt highlights significant gaps in clinical and disease prevention processes of care. This suggests a need to scale up the offer of quit advice to tobacco users by prioritising and integrating tobacco cessation treatments into healthcare programmes in South African hospitals.
